# Supplementary material for: MiR-138-5p Upregulation during Neuronal Maturation Parallels with an Increase in Neuronal Survival
Source: Int J Mol Sci. 2023 Nov 20;24(22):16509. doi: 10.3390/ijms242216509 (PMC10671628; doi:10.3390/ijms242216509)
Supplement: Supplementary file 1 [file ijms-24-16509-s001.zip › Supplementary Figures Barreda-Manso et al IJMS.pdf]

Figure S1:

		W	*p-value*
**Figure 1**	A. miR-138-5p expression	0.95	0.4677
	B. Casp3 expression	0.93	0.3075
	C. pro-CASP-3 expression	0.85	0.101
**Figure 3**	B. pro-CASP-3 expression after LGA stimulation	0.91	0.391
	C. cleaved-CASP-3 expression after LGA stimulation	0.93	0.707
**Figure 4**	B. Caspase activity	0.89	0.109
	C. Neuronal density	0.84	0.001 ***
**Figure 5**	B. Neuronal survival	0.72	0.943
	C. Neuronal death	0.98	0.987
	D. Neuronal density	0.8	0.00028 ***
**Figure 6**	Diferences between treatments at 1 d	0.82	0.111
	Diferences between the two maturation times	0.82	0.015 **

Figure S1. Normality analysis of the studied data. The table show the W and *p*-value of the statistical analyzes of normality performed on the data of the exposed results. ** and *** denote significant differences ($p<0.01$ and $p<0.001$, respectively) and, therefore, absence of normality.

Figure S2:

A

miR-138-5p	1 d	4 d	7 d	14 d	18 d
**mean ΔCt**	2.71 ± 0.80	2.07 ± 0.77	1.00 ± 0.68	0.66 ± 0.80	0.75 ± 0.80
**$2^{-\Delta\Delta Ct}$**	1.00	0.64	0.31	0.24	0.26
**(95% CI)**		(0.20 ; 2.02)	(0.10 ; 0.97)	(0.08 ; 0.76)	(0.08 ; 0.81)

ANOVA	df	F value	p-value
**exp**	2	88.70	p < 0.001
**days**	4	63.29	p < 0.001

Tukey test p-value		
	**4 d - 1 d**	n.s.
	**7 d - 1 d**	p < 0.001
	**14 d - 1 d**	p < 0.001
	**18 d - 1 d**	p < 0.001

B

Casp3	1 d	4 d	7 d	14 d	18 d
**mean ΔCt**	14.28 ± 1.47	15.01 ± 1.64	14.88 ± 1.60	16.38 ± 1.33	16.87 ± 1.59
**$2^{-\Delta\Delta Ct}$**	1.00	1.66	1.51	4.29	6.02
**(95% CI)**		(0.15 ; 18.11)	(0.14 ; 16.47)	(0.39 ; 46.85)	(0.55 ; 65.71)

ANOVA	df	F value	p-value
**exp**	2	36.42	p < 0.05
**days**	4	6.86	p < 0.001

Tukey test p-value		
	**4 d - 1 d**	n.s.
	**7 d - 1 d**	n.s.
	**14 d - 1 d**	p = 0.05
	**18 d - 1 d**	p < 0.05

C

miR-138-5p	1 day		14 days	
	**Ctr**	**LGA**	**Ctr**	**LGA**
**mean ΔCt**	2.57 ± 0.67	2.81 ± 0.81	1.87 ± 0.29	1.85 ± 0.11
**$2^{-\Delta\Delta Ct}$**	1.00	0.84	1.00	1.02
**(95% CI)**	(0.39 ; 2.58)	(0.31 ; 2.70)	(0.55 ; 1,82)	(0.37 ; 2.81)

Figure S2. Quantification and analysis tables for the RT-qPCR analysis of Figures 1 and 6. (A) and (B) Tables show the microRNA and mRNA expression of miR-138-5p and Casp3 as the mean ΔCt and the fold change $2^{-\Delta\Delta Ct}$ (values and limits in folds of the 95% CI) of four independent experiments per time. Statistical analysis was carried out through two-way ANOVA and Tukey post-hoc tests. (C) The table shows the expression of miR-138-5p as the mean ΔCt and the fold change $2^{-\Delta\Delta Ct}$ (values and limits in folds of the 95% CI) of three independent experiments per time.
