# Supplementary figures and images for: MiR-138-5p Upregulation during Neuronal Maturation Parallels with an Increase in Neuronal Survival
Source: Int J Mol Sci. 2023 Nov 20;24(22):16509. doi: 10.3390/ijms242216509 (PMC10671628; doi:10.3390/ijms242216509)

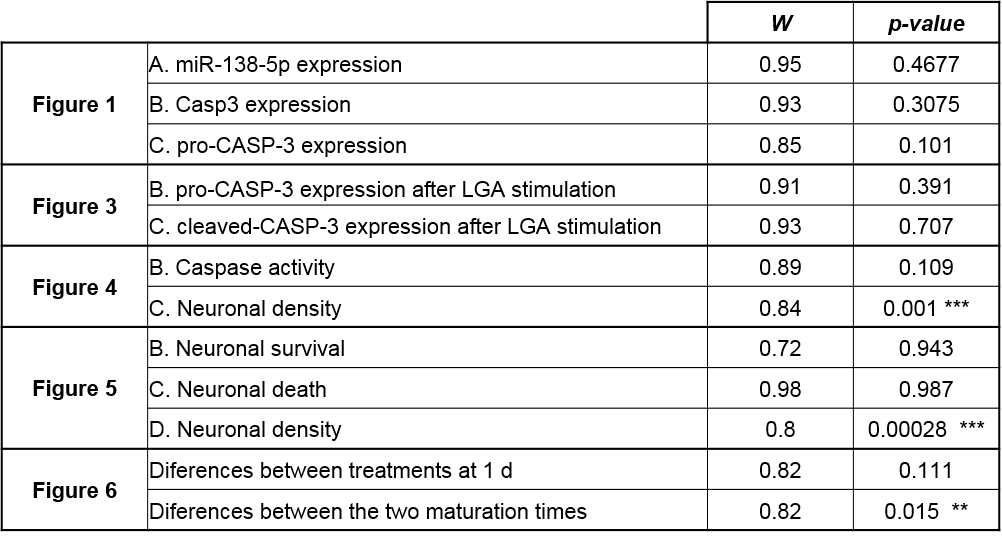

Supplement: Supplementary file 1 [file ijms-24-16509-s001.zip › Figure S1 Barreda-Manso et al.tif]

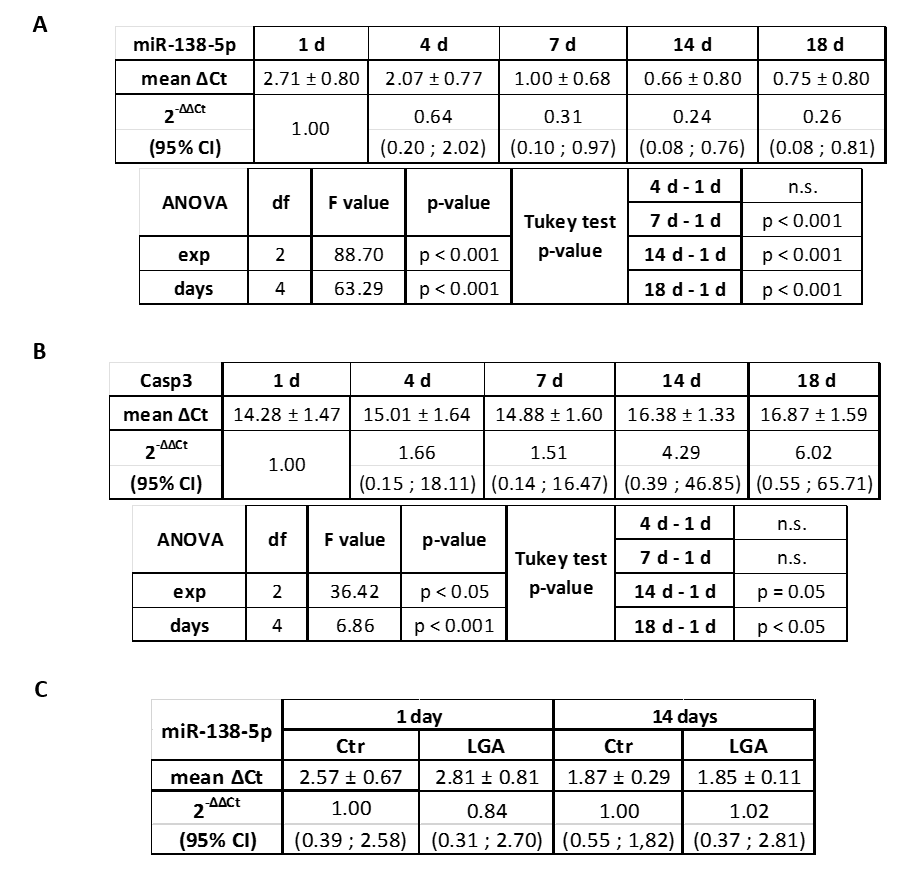

Supplement: Supplementary file 1 [file ijms-24-16509-s001.zip › Figure S2 Barreda-Manso et al.tif]
